# *In Vivo* Near Infrared Virtual Intraoperative Surgical Photoacoustic Optical Coherence Tomography

**DOI:** 10.1038/srep35176

**Published:** 2016-10-12

**Authors:** Donghyun Lee, Changho Lee, Sehui Kim, Qifa Zhou, Jeehyun Kim, Chulhong Kim

**Affiliations:** 1 Future IT Innovation Laboratory, Department of Creative IT Engineering, Pohang University of Science and Technology (POSTECH), 77 Cheongam-ro, Nam-gu, Pohang, Gyeongbuk, 37673, Republic of Korea; 2 Department of Ophtalmology and Biomedical Engineering, University of Southern California, Los Angeles, CA 90033, USA; 3 School of Electrical Engineering, Kyungpook National University, Daegu, 41566, Republic of Korea

## Abstract

Since its first implementation in otolaryngological surgery nearly a century ago, the surgical microscope has improved the accuracy and the safety of microsurgeries. However, the microscope shows only a magnified surface view of the surgical region. To overcome this limitation, either optical coherence tomography (OCT) or photoacoustic microscopy (PAM) has been independently combined with conventional surgical microscope. Herein, we present a near-infrared virtual intraoperative photoacoustic optical coherence tomography (NIR-VISPAOCT) system that combines both PAM and OCT with a conventional surgical microscope. Using optical scattering and absorption, the NIR-VISPAOCT system simultaneously provides surgeons with real-time comprehensive biological information such as tumor margins, tissue structure, and a magnified view of the region of interest. Moreover, by utilizing a miniaturized beam projector, it can back-project 2D cross-sectional PAM and OCT images onto the microscopic view plane. In this way, both microscopic and cross-sectional PAM and OCT images are concurrently displayed on the ocular lens of the microscope. To verify the usability of the NIR-VISPAOCT system, we demonstrate simulated surgeries, including *in vivo* image-guided melanoma resection surgery and *in vivo* needle injection of carbon particles into a mouse thigh. The proposed NIR-VISPAOCT system has potential applications in neurosurgery, ophthalmological surgery, and other microsurgeries.

First implemented in the early 20^th^ century, the surgical microscope has revolutionized microsurgery, enhancing the accuracy and the efficiency of procedures in ophthalmology, neurosurgery, vascular surgery, and plastic surgery^1^,^2^,^3^,^4^. Inherently though, the surgical microscope is limited to showing magnified views of the surface of the surgical region, without any subsurface information, including the position of the underlying microvasculature and nerves. This limitation requires surgeons to have extensive training and experience to minimize unexpected adverse events during surgery. Thus, alternative imaging techniques are needed to supply sub-surface cross-sectional information about the region of interest during surgery. Medical imaging techniques such as magnetic resonance imaging (MRI), X-ray computed tomography (CT), and ultrasound (US) imaging have been investigated to complement the surgical microscope during surgery^5^,^6^,^7^. However, these methods are not particularly well suited for intraoperative employment. Variously, they may require ionizing radiation, have poor resolution or low sensitivity, be cumbersome to handle, or require a long time for image processing.

Several optical imaging techniques for evaluating biological tissue offer advantages such as superior spatial and temporal resolution, real-time display, the ability to supply depth-resolved data, and flexible implementation^8^,^9^. These merits nicely compensate for the drawback of the traditional surgical microscope. Photoacoustic microscopy (PAM) is an emerging non-invasive optical hybrid imaging modality that detects US signals generated by pulsed laser irradiation as a result of light absorption and the consequent thermoelastic expansion of targets^10^,^11^,^12^,^13^,^14^. PAM can visualize the subsurface microstructure of biological tissue and provide functional, molecular, and metabolic information in real time^15^,^16^,^17^,^18^,^19^,^20^. Recently, surgical microscopes integrated with PAM systems were demonstrated, operating in the visible and near-infrared (NIR) ranges^21^,^22^. Although these systems showed the feasibility of PAM in real-time needle guiding and drug delivery monitoring, their insensitive contrast to tissue layers made them unable to monitor the movement of surgical instruments at depth or observe the tissue microstructure. Thus, to compensate for these weaknesses, an additional imaging modality is required. Fortunately, given its fundamentally fused optical and US properties, PAM can be easily blended with several other optical imaging and US imaging modalities^23^,^24^,^25^. Among these other modalities, optical coherence tomography (OCT) in particular has been effectively combined with PAM. The two modalities have similar optical schematics and exploit complementary contrasts: PAM provides microvasculature information based on optical absorption contrast, while OCT visualizes microstructure based on optical scattering contrast^26^. Thus, integrating PAM with OCT enables monitoring the movement of surgical instruments and provides microstructural information.

In this paper, we present a near-infrared virtual intraoperative surgical photoacoustic optical coherence tomography (NIR-VISPAOCT) system that combines a commercial surgical microscope, PAM, and OCT, and uses NIR wavelengths for illumination. Because they share the same optical path, PAM and OCT were easily combined to scan the same area at the same time. Our NIR-VISPAOCT system has four remarkable key features: (1) It provides surgeons with real-time, simultaneous, comprehensive, and co-registered biological information such as tumor margin, tissue structure, and a magnified view of the region of interest. (2) It back-projects 2D cross-sectional PAM and OCT images onto the microscopic viewing plane as a simple augmented reality, so surgeons do not have to redirect their sight during surgery. (3) It eliminates disturbance to the surgeon’s vision by scanning the surgical region with near infrared wavelengths (PAM, 1064 nm; OCT, 880 nm). (4) It uses a needle type unfocused US transducer to minimize the contact area and has no water tank, making it convenient to monitor surgery. To demonstrate the feasibility of the NIR-VISPAOCT system, we successfully conducted two simulated surgical procedures, melanoma removal and needle guiding.

## Results

### Near-infrared virtual intraoperative surgical photoacoustic optical coherence tomography (NIR-VISPAOCT)

**Figure 1:**
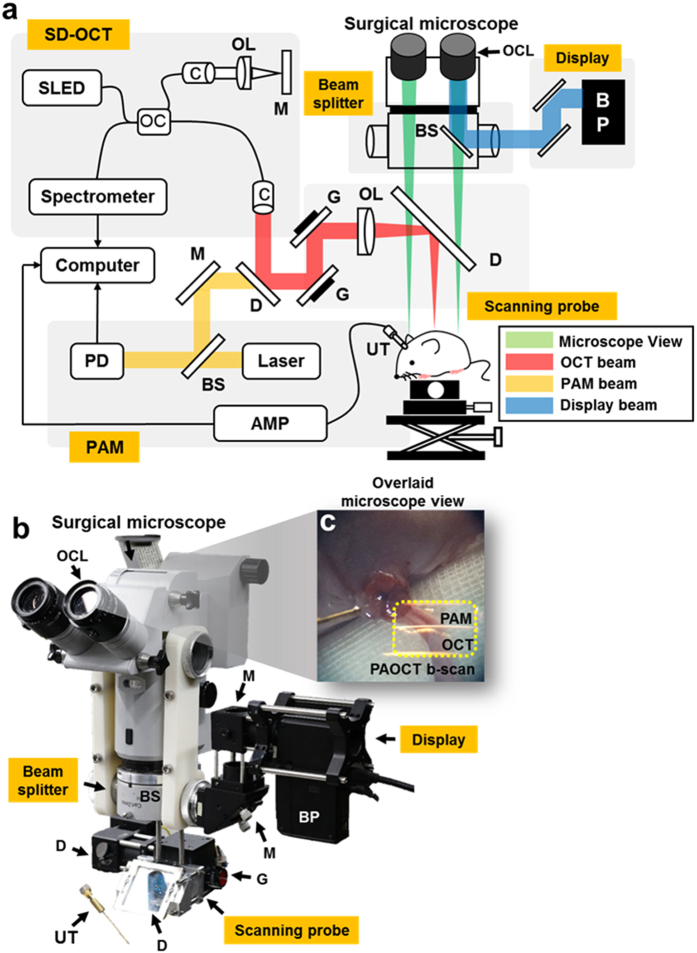
(**a**) Schematic of NIR-VISPAOCT. (**b**) Photograph of the NIR-VISPAOCT probe. (**c**) Surgical microscope image overlaid with B-scan PAM and OCT images. SLED, superluminescent diode; C, collimator; M, mirror; G, galvanometer; OL, objective lens; OC, optical coupler; BS, beam splitter; AMP, amplifier; UT, ultrasound transducer; BP, beam projector; OCL, ocular lens; PD, photodiode; D, dichromic mirror; OCT, optical coherence tomography; and PAM, photoacoustic microscope.

Figure 1a,b show a schematic and a photograph of the NIR-VISPAOCT system. The system is composed of three optical imaging devices (an optical resolution PAM (OR-PAM) system, a spectral-domain OCT (SD-OCT) system, and a conventional surgical microscope) and three customized parts (a scanning probe, an augmented reality display device, and a beam-splitting device). The implementation of the NIR-VISPAOCT system is detailed in the Methods section. Briefly, the OR-PAM and SD-OCT systems’ light sources are combined via a dichroic mirror, passed through the same scanning device, and sent along the same optical path. Therefore, the cross-sectional PAM and OCT B-scan images, with a display rate of 23 Hz, are concurrently acquired and displayed over the same scanning range. Additionally, the fields of view (FOVs) of the cross-sectional PAM and OCT B-scan images are 6 mm × 1.9 mm along the X and Z axes, respectively, and the FOVs of the volumetric PAM and OCT images are 6 mm × 6 mm × 1.9 mm along the X, Y, and Z axes, respectively. To provide significant convenience to surgeons, we improved the PA signal acquisition method and the PAM and OCT image visualization methods. First, we acquired the PA signal with a homemade needle-type transducer directly contacting the US coupling gel on the sample. Therefore, the US coupling water tank that previously impeded surgical procedures was eliminated, and the transducer contact area near the surgical region was minimized. Second, we back projected the PAM and OCT B-scan images onto a microscope ocular lens (Fig. 1c), using the customized augmented reality display device and the beam splitting device. In this way, surgeons can see the PAM and OCT B-scan images on the microscope’s ocular lens, overlaid on the magnified microscopic view.

### Correction of PAM B-scan image distortion

**Figure 2:**
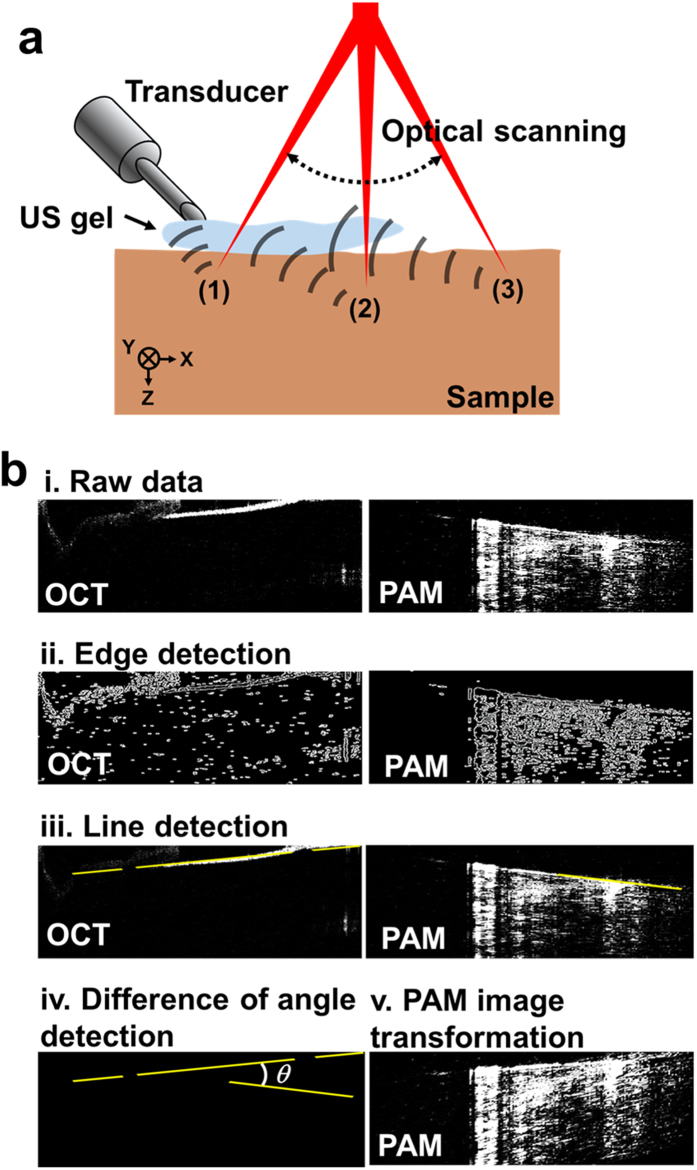
(**a**) Schematic of unfocused needle-type transducer position, optical scanning, and ultrasound wave propagation. (**b**) Correction process for PAM B-scan image distortion. (i) Raw B-scan image data of OCT and PAM before image distortion correction. (ii) Edge detection of OCT and PAM images. (iii) Line detection using Hough transform of OCT and PAM images. (iv) Measuring a difference in angles between the PAM and OCT images. (v) Transformed PAM image.

To detect PA signals with optical scanning, we obliquely positioned the unfocused needle-type transducer at an approximate 45 degree angle with respect to the target sample, as shown in Fig. 2. Therefore, the times of arrival of the PA signals detected near the transducer location (point (1) in Fig. 2) and far from the transducer location (points (2) and (3) in Fig. 2) were different. As a result, PA B-scan images along both axial and lateral directions were distorted compared to the OCT images (Fig. 2bi). To compensate for this distortion, we corrected the PAM B-scan images based on the OCT B-scan images. The correction procedure had four steps: (1) acquisition of PAM and OCT B-scan images of a straight needle, (2) edge and line detection in both PA and OCT B-scan images, (3) calculation of the angle between the two lines extracted from the PAM and OCT B-scan images, and (4) transformation of the PAM B-scan image. In detail, we defined the edges of the needle in the PAM and OCT images for further reconstruction by Canny edge detection (Fig. 2bii). With the edge information, we fitted a straight line of the needle’s path in both of the PAM and OCT B-scan images, using the Hough transform method (i.e., we determined the straight-line minimum of 10 pixels that fit the straight line (Fig. 2biii)). Then we calculated the slope angle between the two straight lines from the PAM image and the OCT image, *θ*, (Fig. 2biv). Finally, we warped the PAM images according to the calculated angle, *θ*, (Fig. 2bv). As a result, the distorted axial position of the PAM images was compensated for.

### Spatial resolution of NIR-VISPAOCT

**Figure 3:**
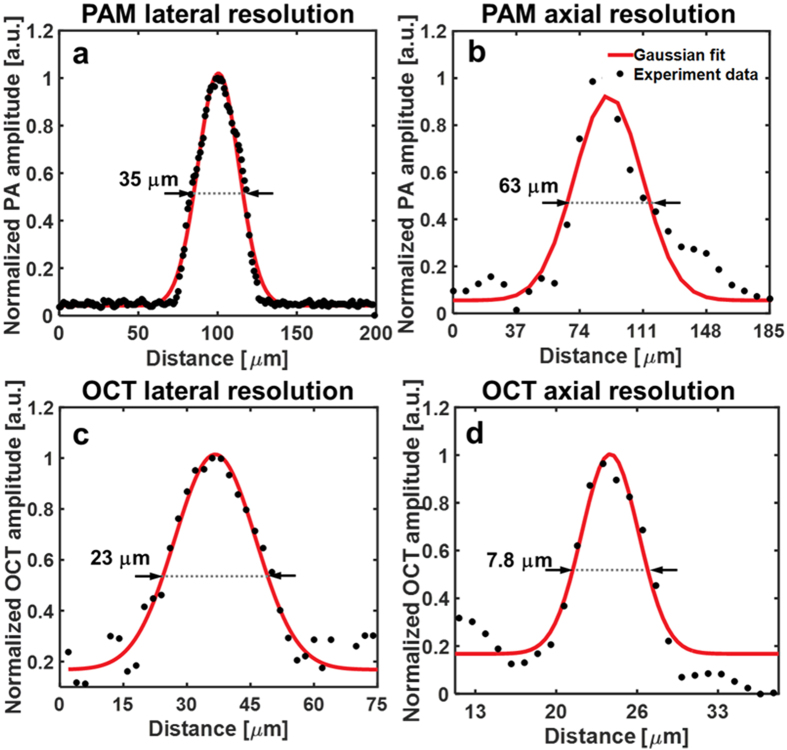
Spatial resolution measurement of the NIR-VISPAOCT system (**a,b**) PAM lateral and axial resolution profile and Gaussian fitting graph, respectively. (**c,d**) OCT lateral and axial resolution profile and Gaussian fitting graph, respectively.

The spatial resolutions of PAM and OCT in the NIR-VISPAOCT system were measured by imaging a carbon fiber and a mirror, respectively. The experimental data were fitted by a Gaussian function, and the full width at half maximum (FWHM) of the fitted Gaussian function was considered to be the spatial resolution. The measured lateral and axial resolutions of the PAM were 35 μm and 63 μm (Fig. 3a,b), where the theoretical lateral and axial resolutions were 32 μm and 57 μm. The lateral and axial resolutions of the OCT were 23 μm and 8 μm (Fig. 3c,d), where the theoretical lateral and axial resolutions were 16 μm and 7 μm. Thus, the experimental result well matched the theoretical values.

### *In vivo* simulated melanoma resection surgery monitoring with NIR-VISPAOCT

**Figure 4:**
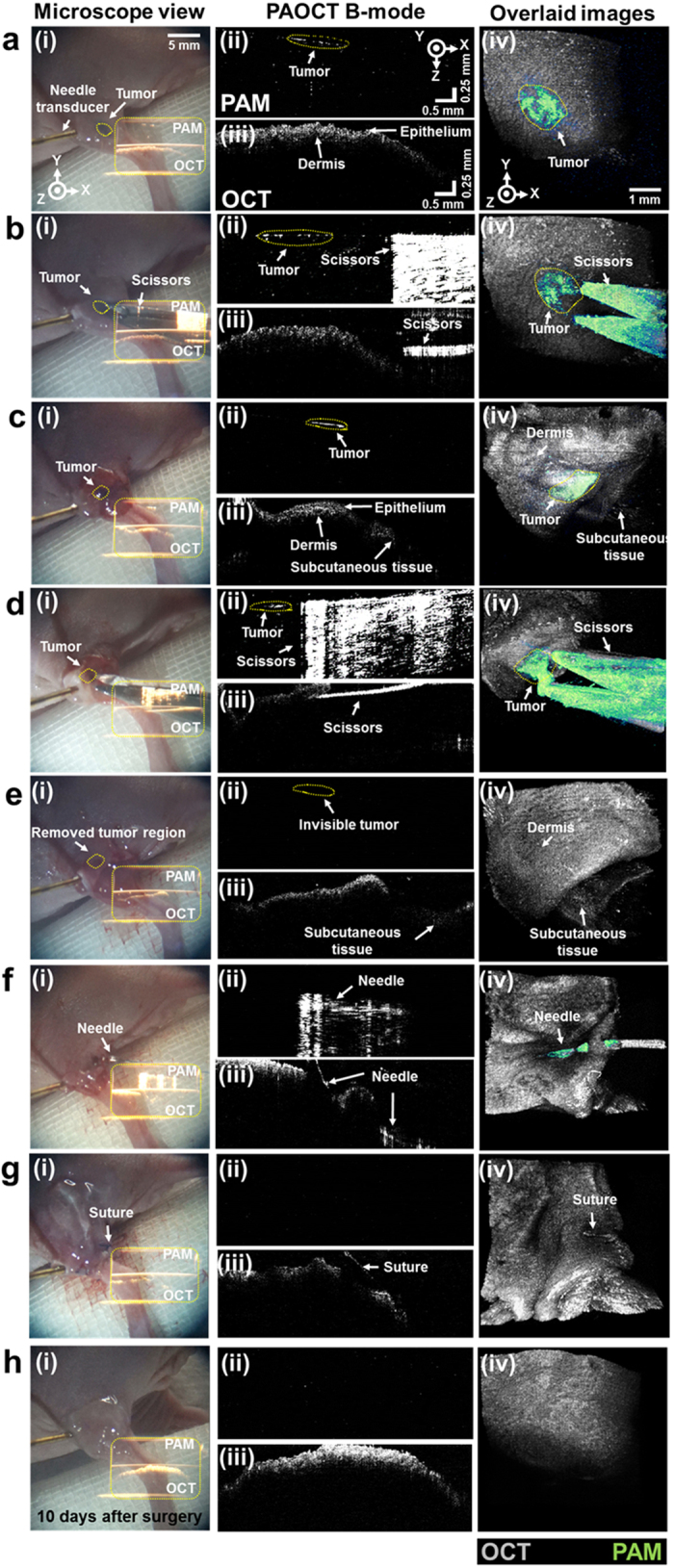
*In vivo* monitoring melanoma resection and suturing surgery process in a melanoma bearing mouse, visualized with the NIR-VISPAOCT system. (**a**) Melanoma detection. (**b,c**) Guiding the surgical instrument near the melanoma and monitoring skin opening. (**d,e**) Melanoma resection. (**f,g**) Suturing. (**h**) Verification of complete melanoma resection 10 days after surgery. (**i**) Screen shot of the surgical microscope ocular lens view. (ii,iii) PAM and OCT B-scan images projected on (i), (ii, PAM; iii, OCT). (iv) PAM and OCT overlaid *en face* images.

We conducted simulated *in vivo* surgeries to demonstrate the feasibility of the NIR-VISPAOCT system. First, we demonstrated an *in vivo* melanoma resection surgery and monitored the surgical processes via the NIR-VISPAOCT system, as shown in Fig. 4. The first column of Fig. 4 (Fig. 4i) shows microscopic view snapshots through the ocular lens during the simulated surgery procedures. Acquired PAOCT B-scan images were back projected on the microscopic view plane through the ocular lens. Thanks to the invisibility of the NIR light source, optical scanning would not disturb the view from the microscope ocular lens. Figure 4ii,iii show close-up PAOCT B-scan images. Figure 4iv shows overlaid *en face* PAOCT images (OCT images in gray, and PAM images in green) clearly visualizing the melanoma’s location and its boundary, the surrounding biological tissue structure, and the surgical instruments.

The melanoma resection surgery was followed by four additional analytic procedures. First, the melanoma imaging and detection were processed. As shown in Fig. 4a, the PAM B-scan image provided boundary information about the melanoma (Fig. 4aii), which did not appear in the OCT B-scan image (Fig. 4aiii). Additionally, the overlaid *en face* PAOCT image clearly shows the melanoma location and its boundary under the skin (Fig. 4aiv). In the second procedure (Fig. 4b–e, Supplementary Video S1), we conducted skin incision and melanoma resection with surgical scissors. As shown in Fig. 4b–d, PAM B-scan images poorly depicted the scissors’ depth information (Fig. 4bii–dii), due to the poor axial resolution of PAM and the high reflection signal from the surface of the scissors. However, during skin incision and the melanoma resection, OCT B-scan images could visualize the correct depth and the scissors’ incision process accurately, without additional damage to deep tissue (Fig. 4biii–diii). The PAM B-scan image (Fig. 4eii) could immediately confirm that the melanoma was successfully removed. The melanoma beneath the skin was also identified in the overlaid *en face* PAOCT image (Fig. 4eiv). In the third procedure (Fig. 4f, Supplementary Video S2), skin suturing was successfully conducted using PAOCT B-scan images to guide the needle movement (Fig. 4fii,fiii). Even when the PAM B-scan images were distorted by low axial resolution and an overly fluctuating PA signal, the OCT B-scan images could monitor the needle movement and tissue condition. Finally, to validate complete melanoma excision, we inspected the removed melanoma area immediately after and 10 days after suturing, using the NIR-VISPAOCT system (Fig. 4g,h). As shown in the PA B-scan image (Fig. 4hii) and the overlaid *en face* PAOCT image (Fig. 4hiv), no reoccurrence was observed 10 days after surgery, indicating complete melanoma excision. These results indicate that the NIR-VISPAOCT system is fully able to guide complete surgical procedures such as melanoma imaging and detection, skin incision and melanoma resection, skin suturing, and post-surgery inspection.

### *In vivo* simulated needle guiding and drug delivery with NIR-VISPAOCT

**Figure 5:**
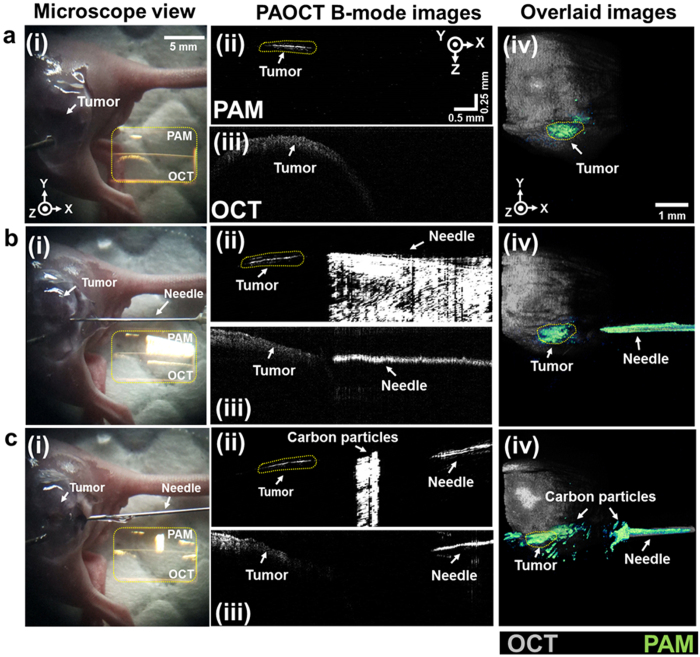
*In vivo* NIR-VISPAOCT-guided needle insertion and carbon particle injection into melanoma bearing mouse. (**a**) Melanoma imaging. (**b**) Needle insertion. (**c**) Carbon particle mixture injection near tumorous region. (i) Screen shot of the surgical microscope ocular lens view. (ii,iii) PAM and OCT B-scan images projected on (i), (ii, PAM; iii, OCT). (iv) PAM and OCT overlaid *en face* images.

Drug injections such as a sub-retinal injection, an intra-articular injection, or an anti-cancer drug injection require delicate and accurate control of the needle to deliver the drug to the targeted site without damaging organs or blood vessels. Accurate delivery of anti-cancer drugs and therapeutic drugs to cancer sites is especially important to minimize damage to normal tissue and maximize the therapeutic effect. Using the NIR-VISPAOCT system as shown in Fig. 5 and Supplementary Video S3, we have performed *in vivo* simulated needle guiding and drug delivery, successfully guiding the needle and delivering a mixture of carbon particles and US gel to a melanoma region. As shown in Fig. 5a, enlarged microscopic images with overlaid PAOCT B-scan images were used to observe the condition of the melanoma region. Both the PAM B-scan (Fig. 5aii) and *en face* PAM (Fig. 5aiv) images successfully visualized the boundary of the melanoma. Figure 5b shows the guiding of the needle, monitored by PAOCT images. Although the PAM B-scan image (Fig. 5bii) shows the needle’s progress toward the melanoma, the OCT B-scan image (Fig. 5biii) allowed guiding the needle with a high axial resolution. Figure 5c shows the successful delivery of the carbon mixture to the melanoma region, monitored by PAOCT images. Because of carbon mixture’s black color, the PAM B-scan (Fig. 5cii) and overlaid *en face* PAOCT (Fig. 5civ) images clearly delineate carbon particles near the melanoma. PAOCT’s complementary B-scan images distinctly visualized the melanoma location, the skin surface, the needle’s relative position, and the location of the injected carbon mixture.

## Discussion

The NIR-VISPAOCT system was developed by integrating a conventional surgical microscope with multimodal optical imaging devices (OR-PAM and SD-OCT) which utilize NIR wavelengths. Based on optical absorption and optical scattering, the system simultaneously provided a magnified view of the surgery region as well as comprehensive subsurface information such as biological micro-scale tissue structure, the depth-resolved location of surgical instruments, the melanoma boundary, and the injection area of carbon particles. Of particular note, highly scattering skin layers were clearly visualized in OCT images, and a mostly light absorbing melanoma was clearly shown in PAM images. Although a needle and surgical scissors were shown in both PAM and OCT images, OCT images showed the movement of the needle and surgical scissors more clearly. Moreover, the combination of PAM and OCT allowed compensation for the distortion of PAM B-scan images. Using OCT B-scan images as the standard, the PA signal delays caused by the unfocused acoustic detection were modified. A simple augmented reality device provided an overlaid view of the surgical site’s magnified surface and the PAOCT B-scan images simultaneously via the surgical microscope’s ocular lens. This capability will allow surgeons to focus on the procedure without having to watch another monitor. Furthermore, by using a trigger signal to synchronize PAM and OCT data acquisition and processing, the system achieved real-time PAOCT B-scan image display at 23 frames/sec, which will be a key advantage in clinical use. Another clinical advantage is the use of a needle type transducer, which eliminates the need for a cumbersome water tank: this refined design allowed us to demonstrate incision and suturing of biological tissue before and after melanoma removal. Finally, the feasibility of the NIR-VISPAOCT system in surgical environments was successfully evaluated by conducting simulated *in vivo* surgeries, including a melanoma resection and image-guided injection of carbon particles at a desired location. To more nearly approach true surgical application, we will concentrate on four improvements: (1) developing an optical scanning method that tracks the movement of the surgical instruments to provide accurate subsurface PAOCT information, (2) implementing a visible aiming beam for confirming the scanning position, (3) updating the real-time 3D volumetric image display with a graphics processing unit (GPU)^27^, and (4) adapting a noncontact PA signal detection method for eliminating the gel currently used for propagating PA waves^28^. With these improvements, we expect that the NIR-VISPAOCT system will contribute to enhanced accuracy in microsurgeries such as neurosurgeries, ophthalmological surgeries, dermatological surgeries, and free autologous tissue transfers.

## Materials and Methods

### Optical-resolution photoacoustic microscopy (OR-PAM) and optical coherence tomography (OCT)

As a PA excitation source for OR-PAM, we used a near-infrared pulsed laser system (Teem photonics, SNP-20F-100, France; 20 kHz repetition rate, 0.7 ns pulse width, 1064 nm central wavelength). The laser beam was passed through a 90:10 beam splitter (Thorlabs, CM1-BP108, USA) to provide a trigger beam for a photodiode (Thorlabs, PDA36A-EC, USA) which produced a trigger signal controlled the beam scanning period. The remaining 90% of the laser beam went to an optical scanning probe for sample scanning. For PA signal detection, we used a homemade needle-type US transducer (central frequency, 41 MHz; University of Southern California, USA). The acquired PA signal was amplified by two successive amplifiers (Mini-Circuit, ZFL-500LN+, USA) and digitized by a data acquisition (DAQ) board (National Instruments, PCI-5124, USA). The raw PA data of each pixel along the X axis was processed by Hilbert transformation for envelope detection, and reconstructed to form one PA B-scan image. Volumetric PA images were acquired by moving galvanometer mirrors along the X and Y axes.

The SD-OCT system utilized a NIR continuous wave superluminescent light emitting diode (SLED, Superlum, SLD-351-HP3, Ireland; central wavelength, 880 nm; and bandwidth, 50 nm). The SLED light was passed through a 50:50 fiber coupler (Thorlabs, FC850-40-10-APC, USA), and the two divided fibers were connected to a reference arm and a sample arm. A spectrometer in the SD-OCT system was composed of a collimator (Thorlabs, LA1765-B, USA), an achromatic doublet lens (Thorlabs, AC508-075-B, USA), a transmission type diffraction grating (Wasatch Photonics, 1800 l/mm, USA), and a 12-bit 2048 pixel line scan CMOS camera (Balser, Sprint SPL2048-140K, Germany). A frame grabber (National Instruments, PCIe-1429, USA) was used for interference OCT signal detection and OCT image acquisition. To compensate for the spectrometer’s nonlinearity, we used *k*-linearization^29^.

The OCT laser power and PA excitation energies were <12 mW/cm^2^ and <51 mJ/cm^2^, respectively, much less than American National Standards Institute limit (72 mW/cm^2^ at 860 nm for OCT, and ~100 mJ/cm^2^ at 1064 nm for PAM).

### Near-infrared virtual intraoperative surgical photoacoustic optical coherence tomography (NIR-VISPAOCT)

The NIR-VISPAOCT system consisted of an OR-PAM, an SD-OCT, and a conventional surgical microscope. To provide co-registered and overlaid PAOCT B-scan images on the microscopic view plane, the surgical microscope (Carl Zeiss, OPMI 6-CFC, Germany) was modified with an optical beam scanning probe, an augmented reality display, and a beam splitter. First, a dichromatic mirror (Edmund Optics, #87-045, USA; transmission wavelength, 985~1600 nm) combined the PAM and OCT beams on the same optical path. Then, the combined PAM and OCT beam went to the customized optical beam scanning probe for optical scanning. The beam scanning probe was designed with a 2-dimensioanl galvanometer (Thorlabs, GVS002, USA) for X-Y plane laser scanning, an achromatic doublet lens (Thorlabs, AC254-075-B, USA) for beam focusing at a proper working distance, and a hot mirror (Edmund optics, #43-957, USA; transmission wavelength, 425~675 nm) for reflecting the laser beam to the surgical region and transmitting visible light from the surgical region to the surgical microscope. The homemade augmented reality display device was composed of a beam projector (Optoma, PR320, Republic of Korea; size, 15 × 14 × 7 cm (X, Y, and Z axes, respectively)), and two mirrors to adjust the position of the illuminated PAOCT image on the view plane. Finally, we customized the beam splitting device to overlay the enlarged microscopic view with acquired PAOCT B-scan images, back projected from the display device. For concurrent PAOCT data acquisition and PAOCT B-scan image display, we synchronized the starting timing of galvanometer scanning, OCT data acquisition, and PAM data acquisition by using a trigger signal from the PAM laser source. The general image processing for OCT and PAM, including *k*-domain linearization, fast Fourier transformation, and Hilbert transformation, was conducted in a central processing unit (CPU). All processes were programmed in LabView. A real-time display speed of 23 frames/sec was achieved for visualizing PAM and OCT B-scan images.

### *In vitro* performance test

A carbon fiber (diameter, 6 μm) and an aluminum mirror (Thorlabs, ME1-G01, USA) were used to measure the spatial resolution of the NIR-VISPAOCT system. The lateral and axial resolutions of the PAM were measured by imaging the carbon fiber in water, and the lateral and axial resolutions of the OCT were measured by imaging the carbon fiber and the mirror in free space.

### *In vivo* NIR-VISPAOCT guided surgical operations

All animal experimental procedures were conducted following the laboratory animal protocol approved by the institutional animal care and use committee of the Pohang University of Science and Technology (POSTECH). And all animal experiments were performed in accordance with the National Institutes of Health Guide for the Care and Use of Experimental Animals. The thigh of a BALB/c nude mouse (weight: ~20 g) was hypodermically injected with 2 × 10^5^ B16 melanoma cells. After the melanoma grew to ~4 mm^3^, we conducted *in vivo* simulated surgeries guided by NIR-VISPAOCT. During the surgical simulation, the nude mouse was placed on a customized X-Y-Z linear stage for position adjustment, and anesthetized by vaporized isoflurane (1 L/min of oxygen and 0.75% isoflurane) gas. For simulated melanoma resection surgery monitoring, we used an operating scissors, a forceps, and a suture needle. The simulated melanoma resection surgery was performed in four steps: (1) melanoma imaging and detection, (2) NIR-VISPAOCT-guided skin incision and melanoma resection, (3) skin suturing, and (4) post-surgery monitoring for up to 10 days. During the surgery, NIR-VISPAOCT guided the skin incision, melanoma resection, and suturing. For simulated needle guiding and drug delivery monitoring, we used a 27 gauge injection needle, a syringe, and a mixture of carbon particles (Sigma-Aldrich, carbon–glassy, spherical powder) and US gel. We inserted the needle toward the melanoma in the mouse thigh and delivered the mixture of carbon particles near the tumorous region.

## Additional Information

**How to cite this article**: Lee, D. *et al*. *In Vivo* Near Infrared Virtual Intraoperative Surgical Photoacoustic Optical Coherence Tomography. *Sci. Rep*. **6**, 35176; doi: 10.1038/srep35176 (2016).

## Supplementary Material

Supplementary Information

Supplementary Video S1

Supplementary Video S2

Supplementary Video S3
