# Supplementary material for: In Vivo Near Infrared Virtual Intraoperative Surgical Photoacoustic Optical Coherence Tomography
Source: Sci Rep. 2016 Oct 12;6:35176. doi: 10.1038/srep35176 (PMC5059626; doi:10.1038/srep35176)
Supplement: Supplementary Information [file srep35176-s1.pdf]

In Vivo Near Infrared Virtual Intraoperative Surgical Photoacoustic Optical Coherence Tomography

Donghyun Lee[1†], Changho Lee[1†], Sehui Kim[1], Qifa Zhou[2], Jeehyun Kim[3], and Chulhong Kim[1*]

[1]Future IT Innovation Laboratory, Department of Creative IT Engineering, Pohang University of Science and Technology (POSTECH), 77 Cheongam-ro, Nam-gu, Pohang, Gyeongbuk, 37673, Republic of Korea

[2]Department of Ophtalmology and Biomedical Engineering, University of Southern California, Los Angeles, CA 90033, USA

[3]School of Electrical Engineering, Kyungpook National University, Daegu, 41566, Republic of Korea

[†]These authors contributed equally to this work

Supplementary information

Supplementary Video 1. *In vivo* monitoring melanoma resection in a melanoma bearing mouse, visualized with the NIR-VISPAOCT system.

Supplementary Video 2. *In vivo* monitoring suturing process after melanoma resection, visualized with the NIR-VISPAOCT system.

Supplementary Video 3. *In vivo* NIR-VISPAOCT-guided needle insertion and carbon particle injection into melanoma bearing mouse.
